# Nearly complete mitochondrial genome of *Trichiosoma vitellina* Linné, 1760 (Hymenoptera: Tenthredinidae): sequencing and phylogenetic analysis

**DOI:** 10.1080/23802359.2020.1715860

**Published:** 2020-01-24

**Authors:** Yaping Chen, Meicai Wei, Huilin Yang, Hannan Wang, Gengyun Niu

**Affiliations:** College of Life Sciences, Jiangxi Normal University, Nanchang, China

**Keywords:** Mitogenome, next-generation sequencing, phylogeny, Tenthredinidae, *Trichiosoma*

## Abstract

The nearly complete mitochondrial genome of *Trichiosoma vitellina* is 15,245 bp long. It has the A + T content of 81.6% and contains 13 protein-coding genes (PCGs), 22 tRNA genes, and 2 rRNA genes. Gene rearrangement is present in the mitogenome of *T. vitellina*. All PCGs use standard ATN as start codons, and most PCGs have complete TAN as stop codons. Phylogenetic analysis demonstrates the position of *T. vitellina* in the Tenthredinoidea. This study provides essential data for the conservation genetics of *T. vitellina* and advances the understanding of the phylogeny of Cimbicidae.

*Trichiosoma* Leach, 1817 is a complicated genus of Cimbicidae. About 38 species have been described as valid in the world and 19 of them have been recorded from Europe (Taeger et al. 2010). However, the body color, sometimes as well as body microsculptures, of the individuals of most species is extremely variable. It was believed that there were only 3 or little more really valid species in Europe (Taeger, personal commmunication, 2010). The situation might be the same for the Asian species of *Trichiosoma*. More mitochondrial genomes are necessary for the species recognition and the phylogeny of *Trichiosoma*.

Samples of *Trichiosoma vitellina* (CSCS-Hym-MC0165) were collected near the town of Songjianghe, Jilin, China (40.621°N, 123.092°E) in June 2016. Genomic DNA was prepared in 150 bp paired-end libraries, tagged, and analyzed with the high-throughput Illumina Hiseq 4000 platform. A total of 39,758,468 raw reads were obtained (SRR:10424533). DNA sequences were assembled using MitoZ (Meng et al. 2019). Two contigs were generated by MitoZ. Valid one was verified with two assembly errors. Assembling *T. vitellina* using *trnG* (65 bp) generated by MitoZ and *Leptocimbex linealis* (15,238 bp, unpublished) as references in Geneious Prime 2019.2.1 (https://www.geneious.com) determined that *trnG* connected to *nad3* without any interval. An interval region (31 bp) between *trnH* and *nad4* was found by assembling *T. vitellina* using *nad5* (1714 bp) and *L. linealis* as references. *Labriocimbex sinicus* Yan et al., 2019 and *T. anthracinum* Forsius, 1930 were utilized as references with the mean depth of coverage across the sequences of 2901 and 55208, respectively, to verify the above results. Annotations were generated in the MITOS web server (Bernt et al. 2013) and revised when necessary using the Geneious Prime software.

The nearly complete mitogenome of *T. vitellina* was 15,245 bp long and contained the typical set of 37 genes, two non-coding regions bordered upstream to *trnY* and downstream to *trnQ*, respectively. The gene arrangement of *T. vitellina* was consistent with that of *L. sinicus* and *T. anthracinum.* Among the 13 PCGs, *nad5*, *nad4*, *nad4l*, and *nad1* were encoded on the reverse strand (N-strand) and the rest nine were encoded on the forward strand (J-strand). All started with ATN and terminated with TAN, except for *nad5* which ended with T. Genes *rrnL* (1357 bp, AT% = 84.3%) and *rrnS* (841 bp, AT% = 84.3%) were found at the conserved locations. There were 263 intergenic nucleotides dispersed between 18 gene pairs, ranging in size from 1 to 40 bp. 25 overlapped nucleotides were also found between four gene pairs, with the longest overlap (14 bp) identified between the *atp6* and *cox3*.

Phylogenetic analysis was based on nucleotide sequences of nine unsaturated PCGs (*nad2*, a*tp8*, *nad4l*, and *nad6* were excluded) and two rRNAs of 37 Symphytan species and two Apocritan species. The Bayesian inference and maximum likelihood trees generated identical topologies (Figure 1). In both trees, the monophyly of Tenthredinoidea and of Cimbicidae were well supported. *Trichiosoma vitellina* sequenced in this study was recovered as a sister group of *T. anthracinum*. *Trichiosoma* was the sister group of *Labriocimbex*.

**Figure 1.**
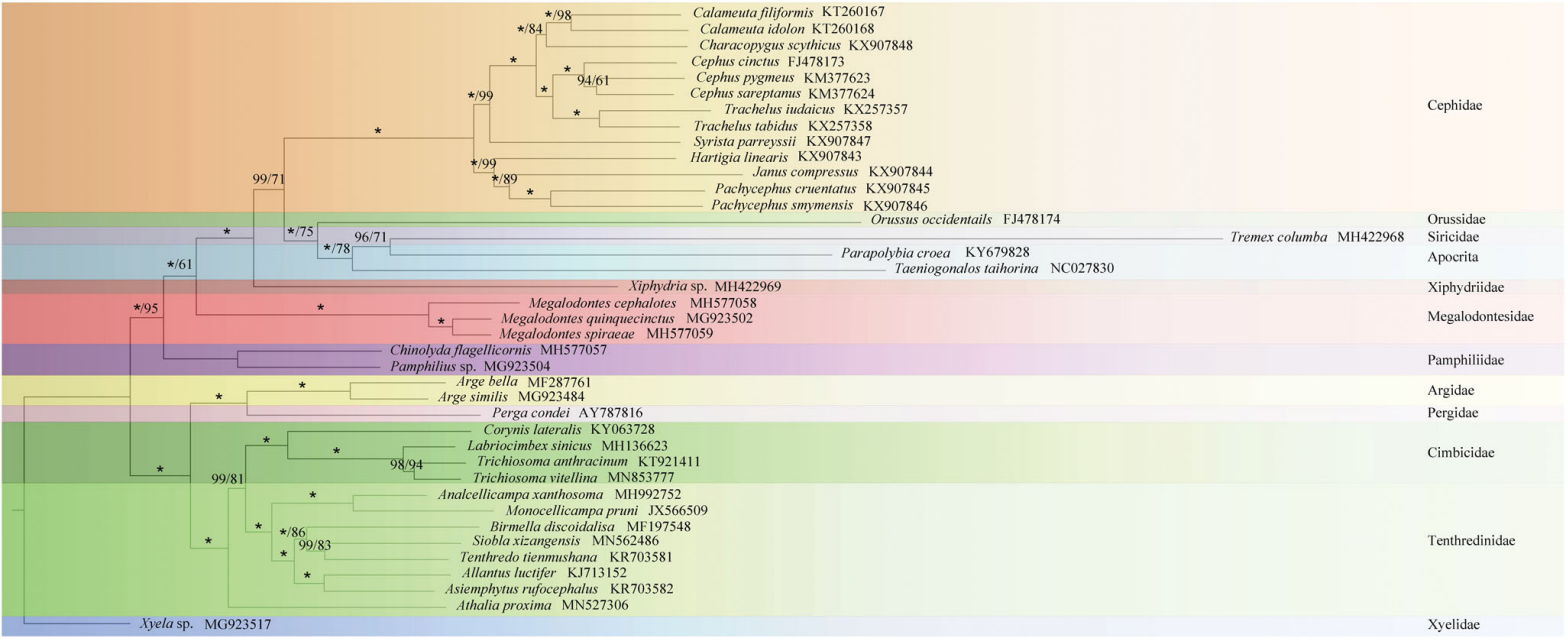
Phylogenetic tree of 39 hymenopteran species based on the combined data of nine unsaturated PGCs and two rRNA genes contained in the mitochondrial genome. Numbers at the nodes are posterior probabilities of the BI analysis (left value, *=100) and bootstrap values of the ML analysis (right value). The GenBank accession numbers are indicated after the scientific names. Branch lengths represent the means of the posterior distributions.
